# Pathophysiological Role of Nucleic Acid-Sensing Pattern Recognition Receptors in Inflammatory Diseases

**DOI:** 10.3389/fcimb.2022.910654

**Published:** 2022-06-06

**Authors:** Norisuke Kano, Guang Han Ong, Daisuke Ori, Taro Kawai

**Affiliations:** Laboratory of Molecular Immunobiology, Division of Biological Science, Graduate School of Science and Technology, Nara Institute of Science and Technology, Ikoma, Japan

**Keywords:** PRR, inflammation, nucleic acid sensing, autoimmune disease, autoinflammatory disease

## Abstract

Pattern recognition receptors (PRRs) play critical roles in recognizing pathogen-derived nucleic acids and inducing innate immune responses, such as inflammation and type I interferon production. PRRs that recognize nucleic acids include members of endosomal Toll-like receptors, cytosolic retinoic acid inducible gene I-like receptors, cyclic GMP–AMP synthase, absent in melanoma 2-like receptors, and nucleotide binding oligomerization domain-like receptors. Aberrant recognition of self-derived nucleic acids by these PRRs or unexpected activation of downstream signaling pathways results in the constitutive production of type I interferons and inflammatory cytokines, which lead to the development of autoimmune or autoinflammatory diseases. In this review, we focus on the nucleic acid-sensing machinery and its pathophysiological roles in various inflammatory diseases.

## Introduction

The innate immune system plays an important role in pathogen recognition, the production of inflammatory cytokines and type I interferons (IFNs), and the effective activation of adaptive immunity, which eliminates pathogens to restore host homeostasis. Innate immune cells, such as macrophages and dendritic cells, express pattern recognition receptors (PRRs) that recognize pathogen-associated molecular patterns, such as bacterial or viral nucleic acids, as well as danger signals released by host in response to cytotoxic damage, termed damage-associated molecular patterns (DAMPs) (Janeway and Medzhitov, 2002; Roh and Sohn, 2018). PRRs include toll-like receptors (TLRs), RIG-I-like receptors (RLRs), cytosolic DNA sensor (cyclic GMP-AMP synthase [cGAS]), and absent in melanoma 2 (AIM2)-like receptors (ALRs). Once activated, PRRs activate their corresponding downstream signaling pathways, leading to the induction of innate immune and inflammatory responses *via* the production of pro-inflammatory cytokines and type I IFNs (Junt and Barchet, 2015). However, the inflammation caused by aberrant PRR activation is harmful to the host. PRRs also recognize self-derived nucleic acids (NAs) to induce undesired inflammatory responses, resulting in autoinflammatory and autoimmune diseases (Schlee and Hartmann, 2016). To prevent such unexpected activation, host cells have a variety of strategies to discriminate between self- and non-self-NAs. In this review, we summarize the recent progress in NA sensing by PRRs and their relevance in various diseases.

## NA Sensing by PRRs

**Figure 1:**
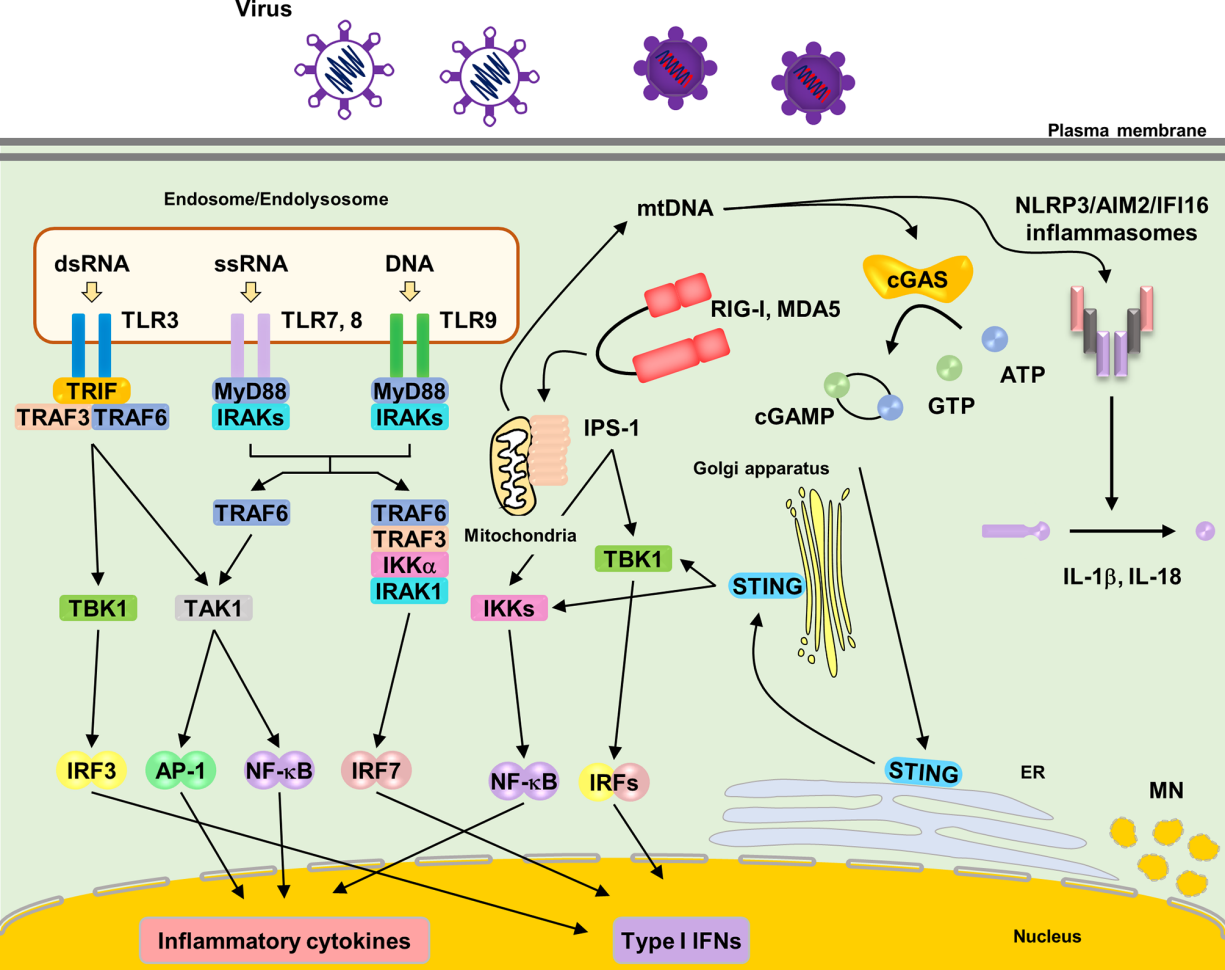
Localization and signaling pathways of nucleic acids sensing TLRs. TLR3, TLR7, TLR8 and TLR9 are localized in endosomes or endolysosomes. Upon ligands ligation of each TLRs, TLR7, TLR8 and TLR9 recruit the adaptor molecules MyD88 to activate downstream signaling pathways. MyD88 recruits IRAKs and TRAF6, subsequently activating TAK1. TAK1 activates NF-κB, leading to induction of proinflammatory cytokines. TLR7 and TLR9 induce IRF7 activation by interacting with TRAF3, TRAF6, IRAK1 and IKKα, resulting in the induction of type I IFNs in pDCs. TLR3 recruits TRIF to activate downstream signaling pathways. TRIF recruits TRAF3 and TRAF6, subsequently, activating TAK1 and TBK1, which leads to the induction of proinflammatory cytokines and type I IFNs *via* NF-κB and IRF3 respectively. RIG-I and MDA5 recognize cytosolic RNA from pathogens. RNA binding to RIG-I and MDA5 causes the exposure of its CARDs domain. Activated RIG-I and MDA5 interact with adaptor molecule, IPS-1 (also known as MAVS), leading to oligomerization on the mitochondrial membrane and activating downstream signaling pathway *via* TRAF3, TRAF6, TBK1 and IKKs, which leads to production of proinflammatory cytokines and type I IFNs *via* NF-κB and IRF3, respectively. Cytosolic DNA sensor, cGAS, recognize cytosolic DNA. Upon DNA binding, cGAS synthetases the cyclic dinucleotide cGAMP. cGAMP subsequently binds to STING on the ER and STING traffics to ER-Golgi intermediated compartment and Golgi apparatus, activating downstream signaling pathway *via* TBK1 and IKKs that lead to production of type I IFNs. NLRP3 and AIM2 inflammasome are also activated by cytosolic DNA. NLRP3 senses mitochondrial DNA, leading to the formation of the inflammasome and the release of IL-1β and IL-18. AIM2 also senses cytosolic bacterial DNA, leading to the formation of the inflammasome and the release of IL-1β and IL-18.

TLRs harbor extracellular leucine-rich repeats, a transmembrane domain, and a Toll/IL-1 receptor (TIR) domain, which signal through downstream signaling molecules, such as myeloid differentiation primary response gene 88 (MyD88) and TIR-domain-containing adapter-inducing interferon β (TRIF) (**Figure 1**) (Duan et al., 2022). Among TLRs, TLR3, TLR7, TLR8, and TLR9 sense NAs (Majer et al., 2017). TLRs are predominantly expressed in intracellular compartments, such as endosomes, endolysosomes, and lysosomes, to minimize exposure to self-NAs. TLR3 recognizes double-stranded RNA (dsRNA) derived from dsRNA viruses and single-stranded RNA (ssRNA) viruses (Wang et al., 2004; Hardarson et al., 2007; Zhang et al., 2007; Kawai and Akira, 2008). TLR7 and TLR8 share general ligand specificity for ssRNA derived from RNA viruses (Diebold et al., 2004). TLR7 recognizes guanosine- and uridine-rich ssRNAs, whereas TLR8 recognizes adenosine- and uridine-rich ssRNAs (Lund et al., 2004). TLR9 senses unmethylated cytosine phosphate guanosine (CpG) motif-containing DNA derived from microorganisms (Hemmi et al., 2000). TLR7, 8 and 9 are predominantly expressed in plasmacytoid dendritic cells (pDCs), which abundantly produce type I IFNs during viral infections (Bender et al., 2020). The signaling mechanisms through these TLRs are shown in **Figure 1**.

RLRs, including retinoic acid inducible gene I (RIG-I), melanoma differentiation factor 5 (MDA5), and laboratory of genetics and physiology 2 (LGP2), are cytosolic RNA sensors belonging to a subfamily of the DExD/H box family of helicases (Onomoto et al., 2021). RIG-I and MDA5 have a C-terminal domain (CTD), DExD/H helicase domain, and two N-terminal cysteine aspartic protease recruiting domains (CARDs) (Saito et al., 2007). RIG-I senses relatively short dsRNA (less than 1 kbp), whereas MDA5 senses long dsRNA (> 1 kbp) (Kang et al., 2002). RIG-I can also recognize 5′-triphosphate ssRNA (Onomoto et al., 2021). In unstimulated state, RIG-I and MDA5 form “closed” conformation as an inactivation form. Upon ligand ligation to their CTD, RLRs change to “opened” conformation, resulting in the exposure of CARDs to subsequently interact with an essential adaptor protein localized on the mitochondrial surface, IPS-1 (also known as Cardif, MAVS, VISA) (Sharma et al., 2021). Once activated, IPS-1 oligomerizes into prion-like filaments to activate downstream signaling.

cGAS senses pathogen-derived cytosolic DNA (Sun et al., 2013; Tan et al., 2018). Furthermore, cGAS recognizes the reverse-transcribed DNA derived from RNA viruses. Upon DNA binding, cGAS synthesizes cyclic dinucleotide cGAMP, which contains one 2–5 phosphodiester linkage and a canonical 3–5 linkage, as the secondary messenger. cGAMP subsequently binds to an endoplasmic reticulum (ER)-localized protein, stimulator of interferon genes (STING), leading to its trafficking to the ER–Golgi intermediate compartment and Golgi apparatus (Ishikawa and Barber, 2008). STING activation requires palmitoylation at the Golgi apparatus (Mukai et al., 2016). Following STING trafficking, it interacts with the downstream signaling molecules, TANK binding kinase 1 (TBK1) and I-kappa-B kinases (IKKs).

Upon ligand binding, PRRs activate downstream signaling pathways *via* corresponding signaling molecules and finally induce the production of inflammatory cytokines and type I IFNs through the transcription factors, nuclear factor (NF)-κB and interferon regulatory factors (IRFs) (Bishani and Chernolovskaya, 2021). Furthermore, they activate the mitogen-activated protein kinase signaling cascade (Chen, 2005). IFNs activate the Janus kinase–signal transducer and activator of transcription signaling pathway *via* the type I IFN receptor to induce several IFN-stimulated genes (ISGs), including IRF7, which in turn amplifies type I IFN induction through a positive feedback loop (Kawai and Akira, 2007).

Cytosolic DNA also activates the NLR family pyrin domain containing 3 (NLRP3) and ALRs, such as AIM2 and interferon-inducible protein 16 (IFI16), leading to inflammasome formation (Xiao, 2015). NLRP3 senses oxidized mitochondrial DNA (mtDNA), which is released into the cytosol (Nakahira et al., 2011; Zhong et al., 2018). AIM2 and IFI16 are responsible for cytosolic sensing of DNA viruses (Bhowmik and Zhu, 2021). Once activated, these PRRs promote the activation of caspase-1, which induces the maturation of interleukin (IL)-1β and IL-18, which are released *via* pyroptosis.

## NA-Sensing TLRs and Diseases

The aberrant activation of TLRs has been implicated in several autoimmune and autoinflammatory diseases (**Table 1**). For instance, TLR7, 8 and 9 are activated by immune complexes containing antibodies against NAs or small nuclear ribonucleoprotein (snRNP) in the sera of patients with systemic lupus erythematosus (SLE) (Boulé et al., 2004). Indeed, pro-inflammatory cytokine production disappeared in bone marrow-derived dendritic cells derived from TLR9-deficient mice when exposed to sera. Furthermore, the loss or inhibition of these TLRs alleviated inflammatory conditions, strongly implying the involvement of these TLRs in autoinflammatory diseases (Nishimoto et al., 2020). In addition, multiple single nucleotide polymorphisms (SNPs) in NA-sensing TLRs have been identified in patients with autoinflammation (Dvornikova et al., 2020; Zhang et al., 2021). Although no functional consequences have been discovered for most SNPs, some of these SNPs have been associated with higher expression of respective TLRs in pDCs or autoantibody production, leading to excessive signaling pathway activation and proinflammatory cytokine production in the development of various inflammatory diseases, such as type 1 diabetes mellitus (T1DM), SLE, and Graves’ disease (Dvornikova et al., 2020).

**Table 1:** Nucleic acid-sensing receptor-related diseases.

Type of PRR	Associated diseases	Evidence	References
TLR3	T1D	Constitutive activation of TLR3 signaling by SNPs in TLR3	(Zhong et al., 2018)
TLR7	SLE	Recognitiono of DNA complex and autoantibodies against snRNP	(Xiao et al., 2015)
	MG	Overexpression in PBMCs and tyhmus	(Nishimoto et al., 2020)
TLR8	SLE	Recognitiono of DNA complex and autoantibodies against snRNP	(Xiao et al., 2015)
TLR9	SLE	Recognitiono of DNA complex and autoantibodies against snRNP	(Xiao et al., 2015)
	MG	Overexpression in PBMCs and tyhmus	(Nishimoto et al., 2020)
	NASH	Recognition of DAMPs released from damaged cells	(Eizirik et al., 2009; Nakahira et al., 2011; Rojas et al., 2018)
	Anemia	Acceleration of erythrophagocytosis via TLR9 on RBCs	(Pacheco et al., 2019)
RIG-I	SMS	Constitutive type I IFNs production by SNPs in RIG-I	(Rice et al., 2014; Lam et al., 2021)
MDA5	AGS, SMS, SLE	Constitutive type I IFNs production by SNPs in MDA5	(Funabiki et al., 2014; Rice et al., 2014; Van Eyck et al., 2015; Nishimoto et al., 2016; Saito et al., 2019)
cGAS-STING	AGS	Enhancement of type I IFNs production caused by nucleases mutation	(Lässig and Hopfner 2017; Crow 2011)
	SAVI, COPA	Constitutive type I IFNs production by SNPs in STING	(Chung et al., 2018; Dhir et al., 2018; Crow and Stetson 2021; Song et al., 2022)
NLRP3	CAPS	Constitutive IL-1β production caused by mutation in NLRP3	(Liu et al., 2014; Günther et al., 2015; Nozawa et al., 2017; Luther et al., 2020)
	obecity	NLRP3 activation by circulating mtDNA	(Gattorno et al., 2007; Masters et al., 2009; de Jesus et al., 2015; Watkin et al., 2015; Volpi et al., 2018; Deng et al., 2020)
	T2D		
	NAFLD		
	gout		
	atherosclerosis		
	neurodegeneration		
AIM2	SLE	Excessive production of IFNs by disruption of AIM2 inflammation formation	(Rheinheimer et al., 2017; Rovira-Llopis et al., 2018)
	psoriasis	Release of IL-1b from keratinocyte induced by cytosolic DNA	(Ising et al., 2019)

A relationship between several autoimmune/inflammatory diseases and viral infections has been indicated. Epstein-Barr virus, a herpes virus that activates TLR3, 7 and 9, is linked with SLE, MS, and myasthenia gravis (Severa et al., 2013; Cavalcante et al., 2018; Jog and James, 2020). Viral infection has also been associated with T1DM, in which innate immune activation *via* TLRs, RLRs, and cGAS leads to islet autoimmunity and pancreatic β cell depletion (Eizirik et al., 2009; Tai et al., 2016; Dvornikova et al., 2020). Molecular mimicry and bystander activation are two popular hypotheses to explain the role of viral infection in autoinflammatory diseases, but much remains to be explored (Rojas et al., 2018; Pacheco et al., 2019). TLR9 is involved in various inflammatory and metabolic diseases, such as atherosclerosis, obesity, and nonalcoholic steatohepatitis (NASH) (Nishimoto et al., 2020). In these diseases, TLR9 is activated by various DAMPs, including cell-free DNA in obesity and mtDNA and HMGB1 released from the damaged liver in NASH (Montes et al., 2015; Nishimoto et al., 2016; Saito et al., 2019). Indeed, these diseases can be rescued by loss or inhibition of TLR9, suggesting that TLR9 is involved in these inflammatory diseases (Nishimoto et al., 2020). Interestingly, Lam *et al.* demonstrated that TLR9 expressed by red blood cells (RBCs) mediates acute anemia in COVID-19 patients with viral pneumonia or secondary infection. During sepsis, an increase in circulating mtDNA and RBC-bound mtDNA levels was observed. This binding activates the TLR9 response, resulting in accelerated erythrophagocytosis, and thus, the development of anemia (Lam et al., 2021).

## RLRs and Diseases

Single nucleotide polymorphisms (SNPs) in *DDX58* (encoding RIG-I) and *IFIH1* (encoding MDA5) were found in patients with autoimmune and autoinflammatory diseases, such as SLE, Aicardi-Goutières syndrome (AGS), T1D, psoriasis, and Singleton-Merten syndrome (SMS) (**Table 1**). Since the report on MDA5 G821S mice, multiple mutations in RLRs have been identified in patients with AGS, SLE, and SMS (Funabiki et al., 2014; Rice et al., 2014; Jang et al., 2015; Van Eyck et al., 2015). MDA5 (R779H) mutations were found in patients with SLE, and MDA5 (K337G, L372F, D339V, A452, G495, K720N, R779H, or C) mutations were found in AGS. Mutations in MDA5 (R821S) and RIG-I (C268F and E373A) were found in the SMS. These gain-of-function mutations are mainly located in the conserved helicase domain, causing increased self/cellular RNA binding or constitutive activation of RLRs without viral infection (Funabiki et al., 2014; Lässig and Hopfner, 2017; Ahmad et al., 2018; Dias Junior et al., 2019). Consequently, these patients often show constitutive type I IFN production called “IFN signature”. Phenotypes with IFN signatures are referred to as type I interferonopathies, which were proposed by Crow *et al.* in 2011 (Crow, 2011). Currently, approximately 40 genotypes can be considered to be type I interferonopathies, involving IFN signaling pathways, proteasome systems, and NA metabolic pathways (Crow and Stetson, 2021). Adenosine deaminases acting on RNA 1 (ADAR1), an adenosine to inosine editing enzyme of dsRNA, inhibits AGS development by regulating type I IFN production and response to type I IFN *via* unexpected MDA5 activation (Song et al., 2022). ADAR1 acts in an inhibitory manner against cell death by negatively regulating MDA5-mediated IFNβ production in specific cells, such as neuronal cells. Some patients with AGS have a loss-of-function mutation in ADAR1 and a bilateral striatal necrosis phenotype with high levels of ISG in the peripheral blood (Song et al., 2022). ADAR1 also protects cells from translational arrest and cell death by suppressing protein kinase R (PKR) activation in response to IFNβ (Chung et al., 2018). Patients with hypomorphic mutations in polyribonucleotide nucleotidyltransferase 1 (*PNPT1*) show constitutive activation of the MDA5-dependent signaling pathway. *PNPT1* encodes the enzyme, PNPase, to avoid the accumulation of mitochondrial double strand RNA (mtdsRNA), while loss of PNPase results in Bax-Bak dependent release of mtdsRNA into the cytoplasm, which leads to induction of type I IFNs *via* the MDA5–IPS1 axis (Dhir et al., 2018).

## cGAS–STING Pathway and Diseases

To avoid sensing self-derived NAs, various nucleases are present in the system. Nuclease deficiency can result in accumulation of self-NA, leading to aberrant inflammatory responses (**Table 1**). For example, mice deficient in the 3′ repair exonuclease encoded by *Trex1* show severe systemic lupus erythematosus (SLE)-like symptoms. Mutations in subunits of the RNaseH2 exonuclease complex (*RNASEH2a, RNASEH2b, RNASEH2c*) result in the accumulation of immune-stimulatory DNA damage, leading to the chronic production of IFNs in some patients with SLE and AGS (Günther et al., 2015; Nozawa et al., 2017). SAMHD1 is a deoxynucleotide triphosphate (dNTP) triphosphohydrolase that has been found to be a negative regulator of the dNTP pool. Some patients with AGS have a mutation in SAMHD1 that results in the accumulation of self-NAs and upregulation of IFNs and ISGs (Günther et al., 2015). Interestingly, the enhancement of type I IFN production in AGS cells caused by nuclease mutations is mediated by the cGAS–STING pathway.

Activation of cGAS–STING pathway plays an important role in pathogenesis of alcohol related liver disease (ALD). Activation of the cGAS–STING pathway and ISGs is observed in the livers of patients with ALD or in primary hepatocytes exposed to alcohol. It has been suggested that alcohol exposure elevates intracellular mtDNA level to activate the cGAS–STING pathway, leading to the pathogenesis of ALD (Luther et al., 2020).

Dysregulated STING activation is also associated with diseases, such as STING-associated vasculopathy with onset in infancy (SAVI) and variations in the coatomer protein complex subunit alpha (COPA) gene. Patients with SAVI show a TBK1-dependent IFN signature because STING SAVI variants are continuously located in the Golgi apparatus, causing persistent activation of TBK1–IRF3-dependent signaling (Liu et al., 2014). COPA syndrome is a genetic disorder characterized by immune dysregulation with an IFN signature (Watkin et al., 2015; Volpi et al., 2018). In patients with COPA, STING is continuously localized in the Golgi apparatus, leading to the activation of the TBK1-dependent signaling pathway under sterile conditions. *Copa* ^E241K/+^ mice showed enhanced levels of IFNβ and ISGs. When these mice were crossed with mice carrying the STING loss-of-function mutation or treated with the STING palmitoylation inhibitor, they recovered from the IFN signature, indicating that the pathogenesis of COPA syndrome is associated with STING palmitoylation (Deng et al., 2020). This evidence clearly indicated that the initiation and termination of IFN production should be tightly controlled.

## NLRP3- and ALR-Mediated NA Sensing and Diseases

Gain-of-function mutations in NLRP3 are responsible for several autoinflammatory diseases, collectively termed cryopyrin-associated periodic syndromes (CAPS) (**Table 1**) (de Jesus et al., 2015). These mutations mainly occur in the ATP-binding cassette NATCH domain, suggesting that the mutated NLRP3 inflammasome can be activated without ATP (Gattorno et al., 2007; Masters et al., 2009). The introduction of these mutations into cells induces rapid necrosis-like cell death (possibly pyroptosis), indicating that the NLRP3 inflammasome may be constitutively activated by these mutations (Fujisawa et al., 2007). In patients with CAPS, NLRP3 mutations trigger constitutive production of IL-1β, resulting in fever, neutrophilic urticaria, conjunctivitis, arthralgia, and elevated acute-phase reactants, which can be alleviated *via* IL-1-blocking treatment (de Jesus et al., 2015). Additionally, the NLRP3 inflammasome is involved in diseases, such as obesity, type 2 diabetes, nonalcoholic fatty liver disease, gout, atherosclerosis, and neurodegeneration (Rheinheimer et al., 2017; Mridha et al., 2017; So and Martinon, 2017; Rovira-Llopis et al., 2018; Ising et al., 2019). Multiple studies have shown that circulating mtDNA levels in plasma or synovial fluid are increased in patients with such diseases. Circulating mtDNA is preferentially detected by NLRP3, subsequently activating the NLRP3 inflammasome to release IL-1β and IL-18 (Nakahira et al., 2011; Hou et al., 2013; Zhong et al., 2018; Yuzefovych et al., 2019).

Studies have also linked AIM2 to SLE pathogenesis. Recent studies have suggested that disruption of AIM2 inflammasome formation leads to increased IFNβ production, a hallmark of SLE (Wang et al., 2018; Thygesen et al., 2019). Furthermore, AIM2 expression and DNA methylation at the AIM2 locus are reduced in patients with SLE (Javierre et al., 2010; Yang et al., 2015). These findings suggest that the expression and epigenetic alterations in AIM2 are associated with the development of SLE. Activation of the AIM2 inflammasome by cytosolic DNA in keratinocytes also contributes to psoriasis pathogenesis (Dombrowski et al., 2011). Conversely, the binding of cytosolic DNA by LL-37, an antibacterial peptide, inhibits its activation, thereby decreasing the release of IL-1β in psoriatic skin (Dombrowski et al., 2011). Collectively, these studies highlight self-DNA-sensing AIM2 as a key driver in certain autoinflammatory diseases.

## NA Sensors in Tumorigenesis and Cancer Therapy

PRRs are crucial in the pathogenesis of autoimmune and autoinflammatory diseases as well as cancer development and therapy. For instance, AIM2 contributes to tumor suppression and development. Several reports have indicated that AIM2 has an unknown inflammasome-independent regulatory role in colorectal tumorigenesis. Even though AIM2-deficient mice produce similar levels of IL-1 and IL-18, more colorectal tumors are found in AIM2-deficient mice than in the wild-type mice (Man et al., 2015; Wilson et al., 2015). Additionally, the release of alarmins, such as IL-1 and IL-18, due to AIM2 activation in immunosuppressive pDCs promotes tumorigenesis in lung cancer (Sorrentino et al., 2015). In contrast, a decrease in tumor growth in cutaneous squamous cell carcinoma is observed in the absence of AIM2 (Farshchian et al., 2017). For development of a cancer treatment strategy targeting AIM2, clinicians must take into consideration the role of AIM2 in different types of cancer.

Several types of cancer cells and culturable cancer cell lines show STING-deficient phenotypes, suggesting that STING may play a role in cancer suppression (Xia et al., 2016; Xia et al., 2016; Kitajima et al., 2019). Cancer-immune cell co-culture experiments demonstrated that downregulation of the cGAS–STING pathway in cancer cells induces resistance to immune effectors. The decrease in ISGs, such as the C-X-C motif chemokine ligand 10, in these immune effector cells leads to a decrease in immune cell infiltration to the tumor site (Peng et al., 2015). Furthermore, tumor-derived DNA or cGAMP activates DCs *via* the cGAS–STING pathway, inducing the production of type I IFNs that enhance the cross-presentation ability of DCs and improve tumor clearance *via* adaptive immunity (Diamond et al., 2011; Fuertes et al., 2011). Recent studies have also demonstrated the role of cGAS–STING as a detector of neoplasm-induced processes. During mitotic cell division, chromosome missegregation may result in a chromosome breakage-fusion-bridge cycle, which induces gene amplification and genome instability (Umbreit et al., 2020). This process, termed chromothripsis, leads to rapid accumulation of mutations and increases the risk of cancer development (Zhang et al., 2015; Umbreit et al., 2020). Fortunately, missegregated DNA forms a separate defective nuclear membrane, called the micronuclei (MN) (Liu et al., 2018). Owing to its defectiveness, MN tends to rupture spontaneously, leaking contained DNA to be sensed by cytosolic DNA sensors (Hatch et al., 2013; Miller et al., 2021). Detection of such DNA by cGAS is suggested to be an immune-surveillance mechanism, clearing potential oncogenic mutated cells and suppressing tumor progression in the early stage (Xia et al., 2016; Harding et al., 2017; Mackenzie et al., 2017).

Activation of the cGAS–STING pathway also contributes to cellular senescence. Senescent cells have MN-like structures in the cytosol and cytoplasmic chromatin fragments (CCFs) (Suzuki et al., 2002). When released into the cytoplasm, CCFs activate the cGAS–STING pathway to develop an inflammatory senescence-associated secretory phenotype (Yang et al., 2017; Glück et al., 2017). As a result, senescence is attenuated and cell proliferation is accelerated in cGAS- or STING-deficient mouse embryonic fibroblasts (MEFs) (Yang et al., 2017). Overall, the cGAS–STING pathway might suppress cancer development by inducing cellular senescence and promoting immune surveillance. Activation of the cGAS–STING pathway promotes cellular senescence-inducing conditions, such as oxidative stress, genotoxic, irradiation, and oncogene expression in MEFs, human primary cells, and cancer (Glück et al., 2017; Dou et al., 2017). In addition, the cGAS–STING pathway is essential for the immune-mediated clearance of premalignant senescent hepatocytes in mice (Xue et al., 2007; Dou et al., 2017; Glück et al., 2017). This makes cGAS–STING an interesting target for cancer therapy. Administration of the STING agonist, dimethyloxoxanthenyl acetic acid (DMXAA), in a solid tumor mouse model resulted in tumor elimination (Zhao et al., 2002). Despite being a strong agonist of mouse STING, DMXAA does not activate human STING (Conlon et al., 2013). This is due to the difference in the STING C-terminal region between mouse and human (Gao et al., 2014). To overcome this hurdle, Ramanjulu *et al.* designed linked dimeric amidobenzimidazole (di-ABZI), a small-molecule STING agonist that interacts with human STING to induce the production of STING-mediated cytokines in human peripheral blood mononuclear cells (Ramanjulu et al., 2018). Administration of di-ABZI inhibits colorectal tumor growth and increases the survival rate in mice (Ramanjulu et al., 2018). However, further studies are required to evaluate the clinical efficacy of di-ABZI and DMXAA, which may be used as potential STING agonists in cancer therapy.

## Conclusion

In recent years, it has become clear that innate and adaptive immune responses can be strongly induced by NAs *via* PRRs. Knowledge of PRRs and their adapter molecules has shed light on the pathogenicity of various autoinflammatory/immune diseases and cancer progression. Although the recognition of NAs derived from microorganisms plays a fundamental role in host defense responses, dysregulation of NA sensing leads to aberrant recognition of self-derived DNA, which strongly contributes to the pathogenesis of autoimmune and autoinflammatory conditions. An increased understanding of NA sensing and self/non-self-discrimination mechanisms at the molecular level may facilitate the development of suitable therapies for these diseases. Further studies are needed to unveil the novel components, regulatory mechanisms, and clinical importance of these sensors.

## Author Contributions

All authors conceptualized the framework of this review article, corrected, read, and finalized the article, and approved the submitted version.

## Funding

This work was supported by JSPS KAKENHI Grants-in-Aid for Scientific Research (B) 20H03468 (T. K.), and JSPS KAKENHI Grant-in-Aid for Early-Career Scientists 21K14817 (D.O.). This work was also supported by the Takeda Science Foundation and JST CREST.

## Conflict of Interest

The authors declare that the research was conducted in the absence of any commercial or financial relationships that could be construed as a potential conflict of interest.

## Publisher’s Note

All claims expressed in this article are solely those of the authors and do not necessarily represent those of their affiliated organizations, or those of the publisher, the editors and the reviewers. Any product that may be evaluated in this article, or claim that may be made by its manufacturer, is not guaranteed or endorsed by the publisher.
